# Proteasome-targeted nanobodies alleviate pathology and functional decline in an α-synuclein-based Parkinson’s disease model

**DOI:** 10.1038/s41531-018-0062-4

**Published:** 2018-08-22

**Authors:** Diptaman Chatterjee, Mansi Bhatt, David Butler, Erwin De Genst, Christopher M. Dobson, Anne Messer, Jeffrey H. Kordower

**Affiliations:** 1 Department of Neurological Sciences, Rush University Medical Center, Chicago, IL c60612 USA; 2 Neural Stem Cell Institute, Regenerative Research Foundation, Rensselaer, NY 12144 USA; 3 Department of Biomedical Sciences, University at Albany, Albany, NY 12208 USA; 4 Centre for Misfolding Diseases, Department of Chemistry, University of Cambridge, Cambridge, CB2 1EW UK; 5 Van Andel Research Institute, Grand Rapids, MI 49503 USA

## Abstract

Therapeutics designed to target α-synuclein (α-syn) aggregation may be critical in halting the progression of pathology in Parkinson’s disease (PD) patients. Nanobodies are single-domain antibody fragments that bind with antibody specificity, but allow readier genetic engineering and delivery. When expressed intracellularly as intrabodies, anti-α-syn nanobodies fused to a proteasome-targeting proline, aspartate or glutamate, serine, and threonine (PEST) motif can modulate monomeric concentrations of target proteins. Here we aimed to validate and compare the in vivo therapeutic potential of gene therapy delivery of two proteasome-directed nanobodies selectively targeting α-syn in a synuclein overexpression-based PD model: VH14*PEST (non-amyloid component region) and NbSyn87*PEST (C-terminal region). Stereotaxic injections of adeno-associated viral 5-α-syn (AAV5-α-syn) into the substantia nigra (SN) were performed in Sprague–Dawley rats that were sorted into three cohorts based on pre-operative behavioral testing. Rats were treated with unilateral SN injections of vectors for VH14*PEST, NbSyn87*PEST, or injected with saline 3 weeks post lesion. Post-mortem assessments of the SN showed that both nanobodies markedly reduced the level of phosphorylated Serine-129 α-syn labeling relative to saline-treated animals. VH14*PEST showed considerable maintenance of striatal dopaminergic tone in comparison to saline-treated and NbSyn87*PEST-treated animals as measured by tyrosine hydroxylase immunoreactivity (optical density), DAT immunoreactivity (optical density), and dopamine concentration (high-performance liquid chromatography). Microglial accumulation and inflammatory response, assessed by stereological counts of Iba-1-labeled cells, was modestly increased in NbSyn87*PEST-injected rats but not in VH14*PEST-treated or saline-treated animals. Modest behavioral rescue was also observed, although there was pronounced variability among individual animals. These data validate in vivo therapeutic efficacy of vector-delivered intracellular nanobodies targeting α-syn misfolding and aggregation in synucleinopathies such as PD.

## Nanomedicine: A disease-modifying approach

Two nanobodies – fragments of antibodies – that target the highly conserved protein alpha-synuclein for degradation reduce neurotoxicity in a rat model of Parkinson’s Disease (PD). Misfolded aggregated alpha-synuclein is the major component of the characteristic protein deposits found in PD, Lewy bodies. Previous studies have shown that alpha-synuclein-binding nanobodies containing a peptide sequence that acts as a signal for protein degradation interfere with alpha-synuclein aggregation in neurons. Jeffrey Kordower at Rush University Medical Center in Chicago, USA, and colleagues have used viruses to deliver these nanobodies into the substantia nigra of rats overexpressing alpha-synuclein in this brain region. The nanobodies not only reduced alpha-synuclein aggregation, they also improved motor function. These findings highlight a promising approach for halting the progression of PD and other neurodegenerative diseases characterized by the accumulation of abnormal alpha-synuclein aggregates.

## Introduction

Synucleinopathies are a class of neurodegenerative diseases featuring misfolding and disordered aggregation of the protein, α-synuclein (α-syn). Parkinson’s disease (PD) is the most common of these disorders and is primarily characterized by significant loss of dopaminergic neurons in the substantia nigra (SN) and abrogation of dopaminergic tone along the nigrostriatal pathway.^1,2^ A prominent neuropathological hallmark of PD is the presence of α-syn centric intracellular inclusions throughout the brain, called Lewy bodies and Lewy neurites.^3,4^ Although the precise mechanisms of α-syn-mediated neurotoxicity are still unknown, findings suggest that overexpression of α-syn in concert with encumbered chaperone activity and dysfunctional protein degradation machinery may trigger aggregation and cellular disruption.^5–9^ Furthermore, mounting evidence of direct cell-to-cell transmission of toxic α-syn species lends weight to the theory of prion-like spatiotemporal progression of pathology.^10–12^ Thus, methods to interrupt proteinaceous inclusion formation and proteopathic seeding represent promising paradigms for therapeutic interventions.

Currently, efforts to mediate α-syn toxicity have primarily utilized α-syn-targeting immunoglobulins to neutralize extracellular transmission of propagating species.^13–16^ However, uncovering methods to probe and impede intracellular induction of proteinopathy in the PD brain is a significant challenge for long-term, lasting remediation of α-syn toxicity. Intrabodies, either in single-chain variable fragment form or single-domain immunoglobulin fragments (V_H_, V_HH_, or V_L_), provide a novel therapeutic approach for intracellular targeting of disordered antigens in neurological disease.^17^ Numerous candidate intrabodies have been selected and tested in vitro targeting various species of α-syn, including monomeric, oligomeric and protofibrillar, and fibrillar forms (reviewed by: Bhatt et al.^18^ and De Genst et al.^19^). Previously we have characterized in situ efficacy of two primary aptamer nanobodies, VH14 (otherwise referred to as NAC14)^20^ and NbSyn87,^21^ to interfere with mutant α-syn aggregation events and dampen model system proteostatic burden.^22^

**Fig. 1:**
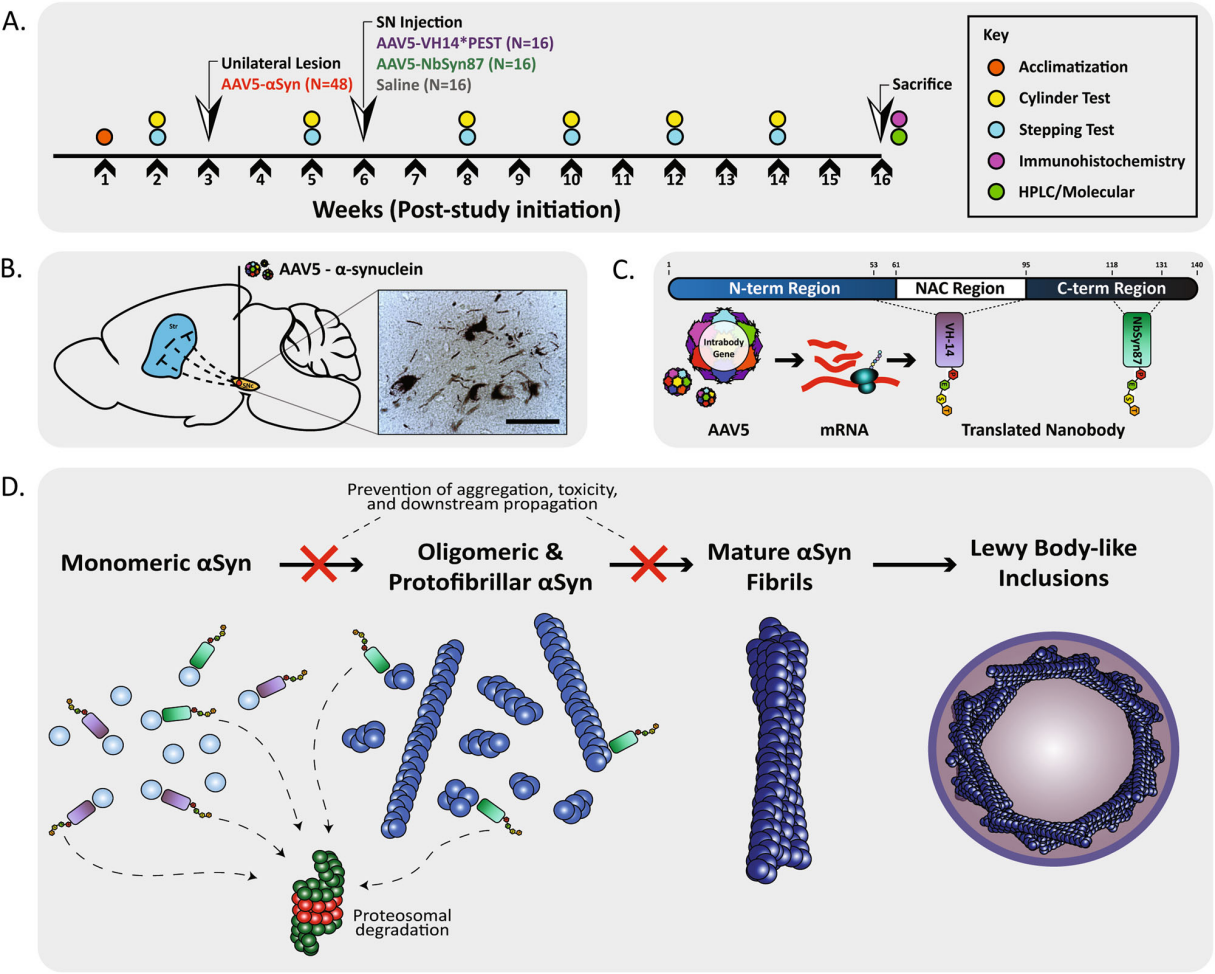
Experimental design and study overview. **a** Study design: genes expressing intrabodies or saline vehicle were delivered by viral vector into animals previously lesioned with an α-syn overexpression vector for observation of treatment effects on behavior and post-mortem analysis of pathology. **b** Model overview of α-syn overexpression via viral vector delivery of human α-syn into the substantia nigra pars compacta (scale bar: B—50μm). **c** Schematic of α-syn protein and target regions for nanobody aptamers VH14*PEST and NbSyn87*PEST. **d** Diagram of the process of α-syn fibril formation, sites of intrabody binding, and intrabody-mediated protein clearance mechanisms

VH14 was screened from a public access, yeast surface display library and has a high-binding affinity for the hydrophobic non-amyloid component region of monomeric α-syn (Fig. 1a, c) found to be critical in fibril formation.^20^ VH14 is a nanobody derived from human origin, making it a prime candidate for evading immunogenicity and inflammatory perturbation in the context of clinical PD. NbSyn87 is a fully camelid nanobody selected from a phage display library.^23^ NbSyn87 belongs to a class of nanobody aptamers that target the C-terminal region of α-syn (Fig. 1c, d) that is known to be the site of post-translational modifications affecting pathogenic triggers of misfolding.^24^ As the C-terminal tail of α-syn remains exposed in oligomeric and protofibrillar species of aggregated complexes, NbSyn87 provides the added benefit of binding to both monomeric and early fibrillar forms of α-syn, although epitope affinity is markedly reduced in advanced maturation stages.^21^ Moreover, NbSyn87 and another nanobody, NbSyn2, which are both specific for the C-terminal region of α-syn, have been shown to inhibit the formation of toxic oligomers in vitro and protect against the toxic action of these oligomers in cell culture models.^25^

Nanobody aptamers independent of stabilizing light-chain fragments are often poorly soluble and prone to aggregation, potentially exacerbating the proteostatic burden in the context of the PD neuraxis. Previously, we have validated enhanced nanobody solubility by engineering a polypeptide tether construct rich in proline, aspartate or glutamate, serine, and threonine (PEST) residues.^22,26,27^ The fusion of the PEST motif decreases the overall net charge and isoelectric point of the protein nanobody improving soluble aptamer expression and effective concentrations in vitro. PEST constructs also act as ubiquitin-independent proteosomal-targeting motifs capable of rapidly degrading bound antigen. We have previously confirmed that the fusion of a PEST degron, derived from a murine ornithine decarboxylase protein, to both VH14 and NbSyn87 efficaciously stalls α-syn aggregation, mediates proteasomal clearance of α-syn, and attenuates proteopathic stress.^22^

Here, we demonstrate in vivo, proof-of-concept efficacy data of two candidate intrabodies, VH14*PEST and NbSyn87*PEST, delivered by gene therapy to a viral α-syn overexpression rodent model of PD. This study validates the proposition that both engineered nanobodies can reduce aggregation of α-syn and limit the expression of α-syn species phosphorylated at Serine-129 (p-S129) correlative of pathological aggregation. Additionally, our data suggest that VH14*PEST α-syn targeting attenuates functional indices reflective of nigrostriatal dopaminergic integrity. These data corresponded with modest preservation in gross motor behavior in the VH14*PEST-treated cohort. Although displaying inferior rescue of striatal dopaminergic output measures in comparison with VH14*PEST, NbSyn87*PEST treatment also showed indications of protected motor function. Collectively, these data provide a platform for further pre-clinical screening of α-syn targeting intrabodies as potential therapeutics for PD and other synucleinopathies.

## Results

### VH14*PEST and NbSyn87*PEST expression reduces pathologic aggregation of α-syn

**Fig. 2:**
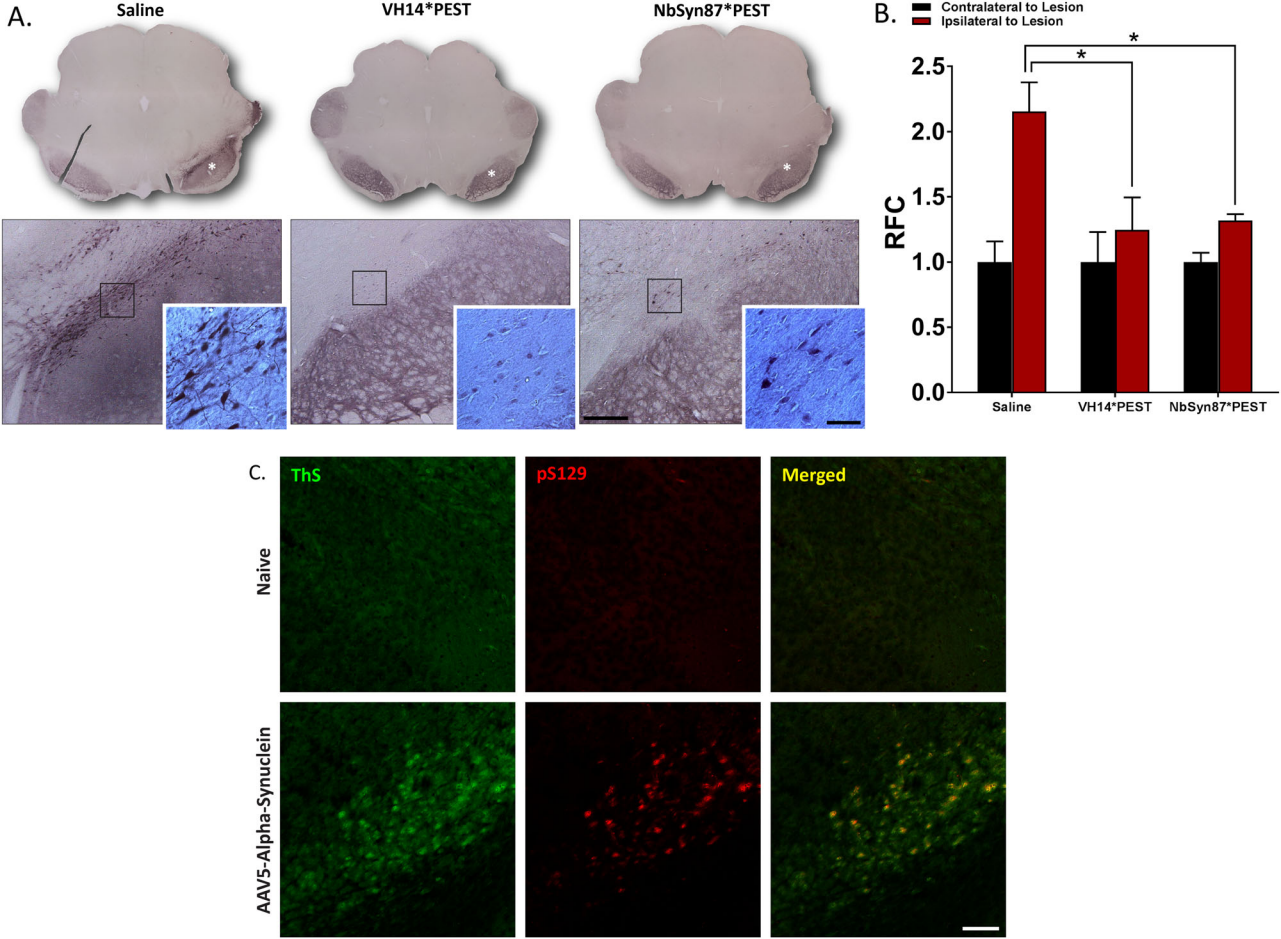
Nanobody expression significantly reduces induced pathological synucleinopathy. **a** Labeling of phosphorylated Serine-129 α-syn species in the substantia nigra reveals reduction of pathological aggregates in both intrabody-treated cohorts compared to saline controls. Intrabody treatment also reduces the overall level of mature Lewy body-like cellular aggregates (inset) (scale bars: 100 and 25 μm). **b** Quantification of optical density measurements of phospho-Serine-129 labeling in the substantia nigra. Both VH14*PEST and NbSyn87*PEST treatment groups reveal significant decreases in densitometric labeling of pathological α-synuclein species. The data were analyzed via one-way ANOVA with Tukey's post hoc comparisons (saline—*n* = 8, VH14*PEST—*n* = 7, NbSyn87*PEST—*n* = 7, all *p < 0.05). **c** Dual immunofluorescent labeling of phosphorylated Serine-129 and Thioflavin-S confirms that phosphorylated residues are representative of aggregated α-syn species (scale bar: 100 μm)

To model α-syn-based proteinopathy, we utilized a viral vector overexpression model of human wild-type α-syn. All subjects received a unilateral injection of an AAV5-α-syn construct directly into the substantia nigra (Fig. 1b). Contralateral hemispheres were not injected and used as controls for lesioned hemispheres. To equilibrate baseline functional performance, lesioned animals were pre-sorted into treatment groups based on baseline behavioral function (Fig. 4). Untreated lesions generated consistent, unilateral Lewy body-like inclusions (Fig. 1b) in the soma and neurites of injected nigral neurons. These protein bodies were resistant to proteinase K digestion, indicating insoluble α-syn inclusions representative of pathologic aggregates (Fig. S2). Three weeks post lesion, animals were injected with viral vectors expressing VH14*PEST or NbSyn87*PEST, or were injected with an equivalent volume of control saline. Histological analysis of post-mortem tissue revealed significant (*p* < 0.05) reduction in labeling of p-S129, as measured densitometrically relative to the unlesioned side (Fig. 2a, b). VH14*PEST and NbSyn87*PEST treatment produces a near twofold reduction of relative pathological aggregate labeling compared to saline treatment of lesions (Fig. 2b). Inclusion formation is markedly reduced in both nanobody-treated groups, with VH14*PEST displaying robust reduction of p-S129 labeling (Fig. 2a, inset). Dual immunofluorescent labeling of p-S129 α-syn and Thioflavin-S validates the labeling of p-S129 residues corresponding with β-sheet aggregation, as is the case in authentic Lewy body pathology (Fig. 2c).

Due to some contralateral background staining observed in p-S129 staining in the SN, we stained for human-specific α-syn species (LB509) to confirm the expression of vector-derived α-syn on the side of lesioning (Fig. S1a). Robust expression of α-syn was predominantly localized ipsilateral to the site of lesion (Fig. S1a). Densitometric measurement of LB509-labeled α-syn in the SN did not reveal statistically significant changes in treatment groups, although there were slight non-significant decreases for both VH14*PEST-treated and NbSyn87*PEST-treated cohorts (Fig. S1b). Taken together, these data demonstrate that both intrabodies reduce α-syn protein concentration at a permissive rate for inhibited proteopathic aggregation but do not excessively clear α-syn species to the point of phenotypic loss of function.

### VH14*PEST halts α-syn-induced pathology afflicting nigrostriatal dopaminergic tone

**Fig. 3:**
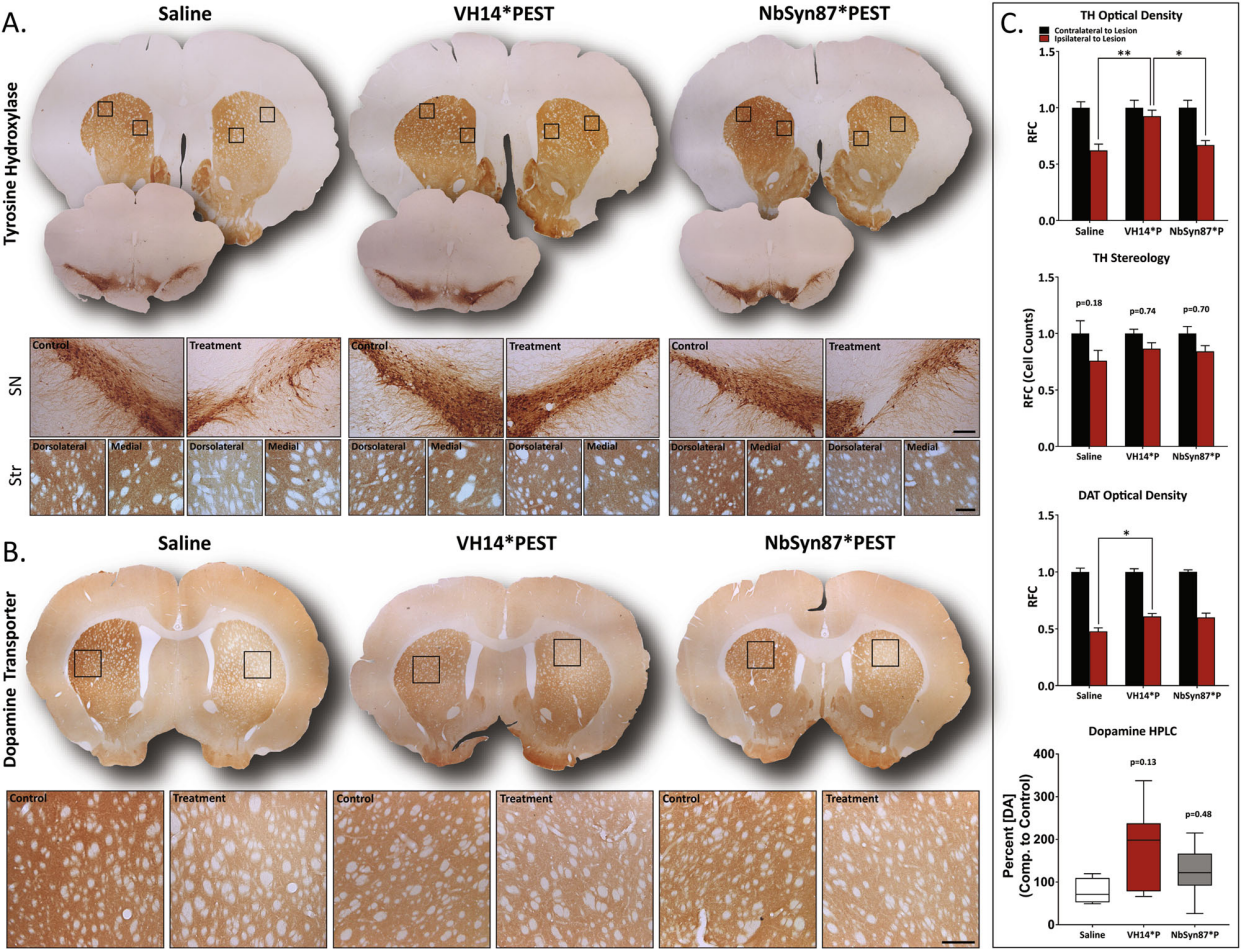
VH14*PEST treatment counteracts insults in nigrostriatal dopaminergic tone induced by synucleinopathy. **a** Assessment of TH^+^ immunoreactivity in the substantia nigra and striatum. VH14*PEST reveals preservation of striatal innervation in comparison to saline-treated and NbSyn87*PEST-treated animals. Evidence of protection is strongest in the dorsolateral striatum (scale bars: 100 and 25 μm). **b** Immunostaining of dopamine transporter (DAT) expression in the striatum shows modest improvement in VH14*PEST treatment animals compared to saline-treated animals (scale bar: 25 μm). **c** Quantification of striatal TH optical density (saline—*n* = 7, VH14*PEST—*n* = 7, NbSyn87*PEST—*n* = 8), stereological cell counts of TH-labeled cells in the substantia nigra (saline—*n* = 7, VH14*PEST—*n* = 8, NbSyn87*PEST—*n* = 7), striatal DAT optical density (saline—*n* = 7, VH14*PEST—*n* = 8, NbSyn87*PEST—*n* = 7), and the concentration of striatal dopamine (saline—*n* = 4, VH14*PEST—*n* = 7, NbSyn87*PEST—*n* = 6). All data were analyzed via one-way ANOVA with Tukey's post hoc comparisons (all *p < 0.05, **p < 0.01, or as indicated on figure)

Given the success of both intrabodies in governing α-syn aggregation, we next aimed to evaluate nigrostriatal pathology central to canonical PD phenotype. Histological analysis of tyrosine hydroxylase (TH) labeling in the striatum revealed significant preservation of synaptic innervation in VH14*PEST-treated animals compared to both saline-treated and NbSyn87*PEST-treated animals (Fig. 3a). TH^+^ deficiency of the lesioned hemispheres was more prominent in the dorsolateral region of the striatum (Fig. 3a). By contrast, relative quantitation of VH14*PEST-treated animals showed a 49% increase in striatal TH^+^labeling compared to saline-treated animals and a 38% increase compared to NbSyn87*PEST-treated animals. Histological analysis of TH labeling in the striatum revealed significant preservation of synaptic innervation in VH14*PEST-treated animals compared to both saline-treated and NbSyn87*PEST-treated animals.

Next, we evaluated the stereological density of TH^+^-labeled cells in the SN (Fig. 3a). In our hands, 4-month post-model induction was insufficient time to see significant loss in nigral dopaminergic neurons in lesioned hemispheres, as has been previously reported.^28^ However, intrabody treatment shows a trend in neuroprotection by one-way analysis of variance (ANOVA) comparison of *p* values between lesioned and contralateral control nigral neuron densities (Fig. 3c). To establish a more complete profile of striatal dopaminergic capacity, we stained sections for dopamine transporter (DAT) expression (Fig. 3b). Consistent with the TH data, the VH14*PEST-treated cohorts showed modest protection of overall DAT expression with a 28% increase in optical density relative to control expression (Fig. 3c). NbSyn87 treatment also ameliorated both TH and DAT expression, but at statistically insignificant levels (Fig. 3a–c). To determine the overall supply of striatal dopamine, tissue of eight animals from each cohort was processed by high-performance liquid chromatography to determine relative dopamine concentrations. Although changes in striatal dopamine supply were statistically insignificant, median dopamine concentration in VH14*PEST-treated animals relative to the control hemispheres was ~3-fold higher than that of the saline-treated group (*p* = 0.13) (Fig. 3c). Individual subjects had dynamic variability in the total dopamine concentrations recorded.

**Fig. 4:**
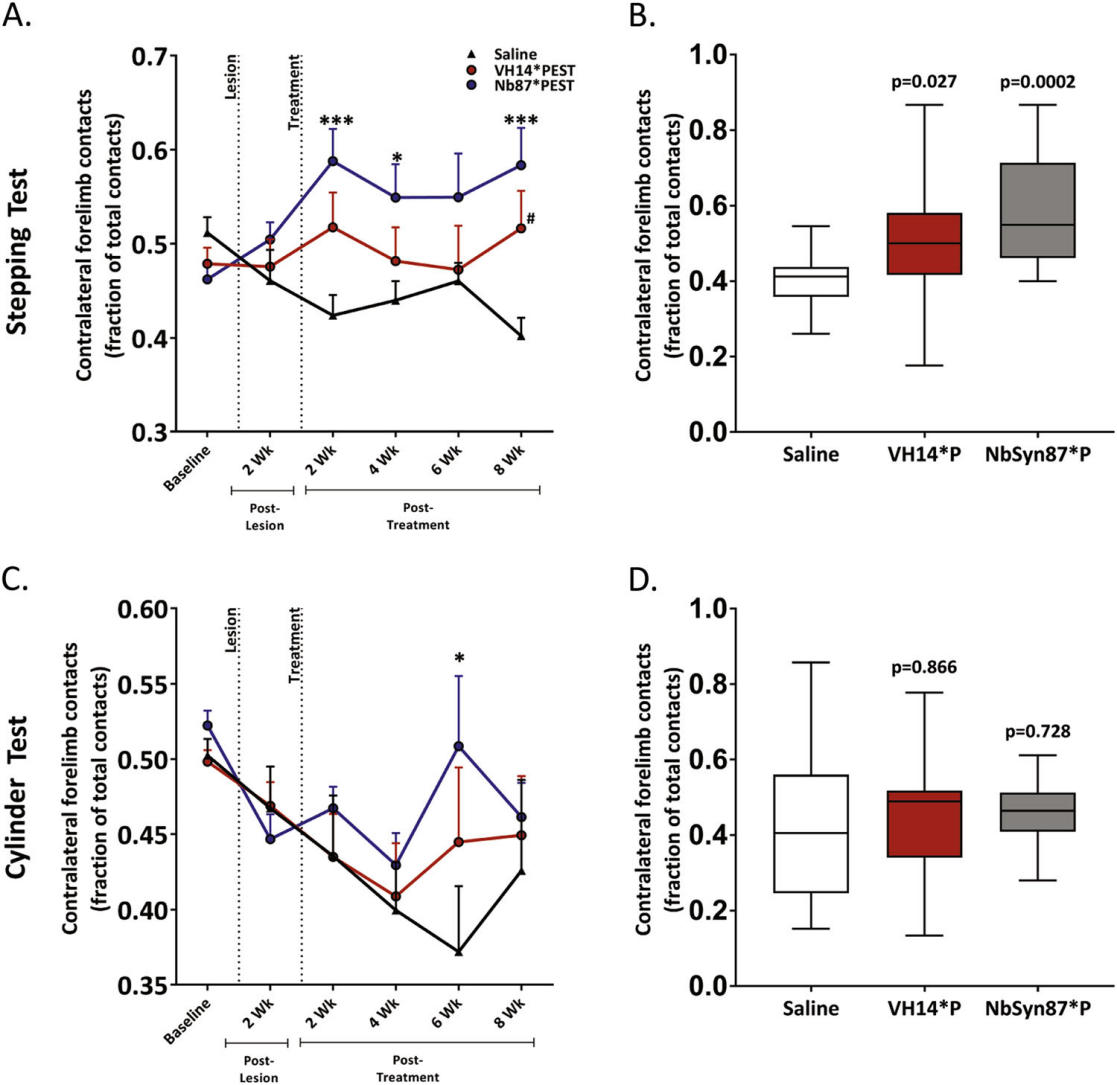
Nanobody treatment induces functional motor recovery. **a** Results of stepping test behavior to assess unilateral forelimb akinesia induced by synucleinopathy and intrabody treatment effects. Both intrabody-treated cohorts improve use of forelimbs contralateral to site of lesion. **b** Box-and-whisker graph of study-endpoint stepping test behavioral performance. **c** Results of cylinder test behavior to assess unilateral forelimb akinesia and intrabody treatment effects. **d** Box-and-whisker plot of study-endpoint cylinder test behavioral performance. All the data were analyzed via two-way ANOVA with Tukey's post hoc comparisons (saline—*n* = 14, VH14*PEST—*n* = 15, NbSyn87*PEST—*n* = 14, all *p* values reported are comparisons with control subjects: *p < 0.05, ***p < 0.001, ^#^*p* < 0.05, or as indicated on the figure. No significant findings were observed in comparison with intrabody-treated cohorts)

To appraise intrabody-mediated changes to nigrostriatal output, we evaluated motor phenotype via two behavioral assays throughout the study: the stepping test and the cylinder test. VH14*PEST-treated animals showed significant (*p* < 0.05) improvement in motor behavior compared to saline-treated control in stepping test performance at the study endpoint (Fig. 4a, b). In contrast to histopathological evaluation of nigrostriatal tone, NbSyn87*PEST-treated animals showed improved rescue of the motor phenotype. NbSyn87*PEST-treated animals displayed significant increases in stepping test contralateral forelimb contacts in comparison to lesioned saline controls from 2-week post lesion through to the study endpoint (Fig. 4a, b). NbSyn87*PEST-treated animals showed less rapid preservation of motor behavior in the cylinder test performance compared to saline controls, only displaying significant improvement 6 weeks post lesion (Fig. 4c, d). In both behavioral assays, no statistically significant differences were found in motor performance between both intrabody-treated cohorts. Individual animal performance in both intrabody cohorts was highly variable, coinciding with the pronounced variance in striatal dopamine levels (Fig. 4b, d). Collectively, these data show VH14*PEST treatment to elicit significant preservation of multiple indices of nigrostriatal health and pathway maintenance in the context of induced synucleinopathy. However, the NbSyn87*PEST-treated group, although showing minimal evidence of dopaminergic rescue, only modestly conserved overall motor function.

### NbSyn87*PEST elicits inflammatory response in lesioned SN

**Fig. 5:**
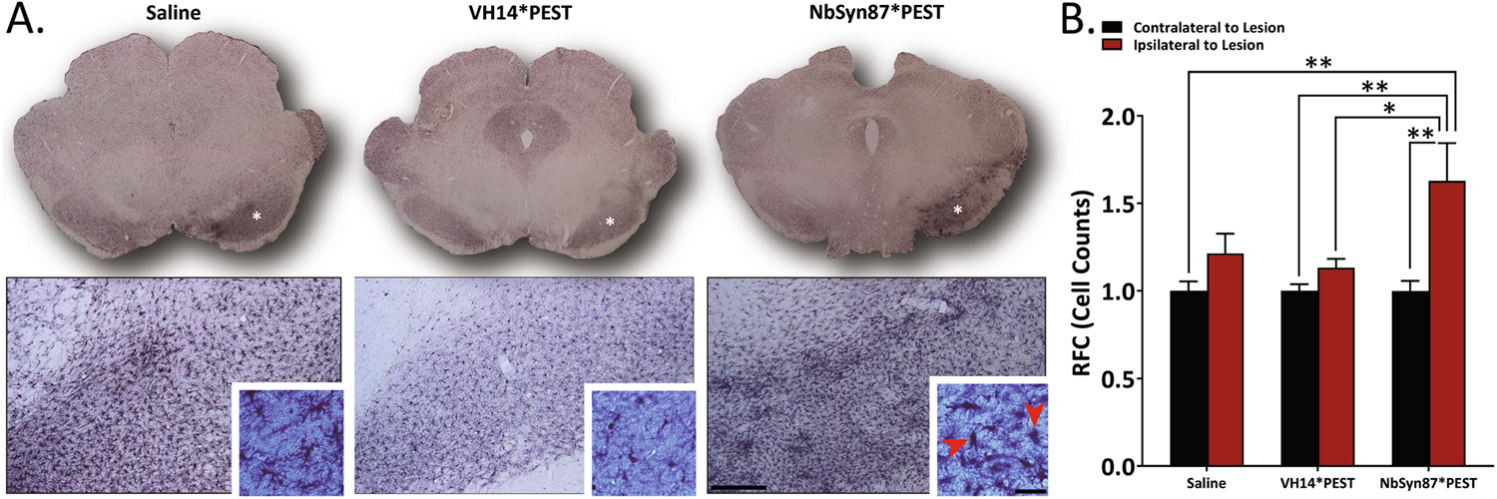
NbSyn87*PEST treatment elicits inflammatory response in substantia nigra. **a** Immunolabeling of Iba-1 in the substantia nigra reveals microglial recruitment and activation. NbSyn87*PEST treatment induces higher levels of microglial clustering to the injected substantia nigra than in VH14*PEST-treated animals. NbSyn87*PEST-treated animals also exhibit higher populations of amoeboid microglia indicating active microglial morphology (red arrow, inset) (scale bars: 100 and 25 μm). **b** Stereological quantification of Iba-1 labeled cells indicates significant increases in microglial recruitment in NbSyn87*PEST, representative of a stronger inflammatory response. Data were analyzed via one-way ANOVA with Tukey's post hoc comparisons (saline—*n* = 6, VH14*PEST—*n* = 8, NbSyn87*PEST—*n* = 7, all *p < 0.05, **p < 0.01)

A critical aspect of gene therapy and immunoglobulin-derived treatment constructs is immunogenicity and a potentiated inflammatory response to treatment. To evaluate potential inflammation, we investigated the recruitment and activity of microglia to sites of injection in the SN. We immunolabeled the microglial calcium-binding protein marker, Iba-1, as a reporter of resident macrophage activation (Fig. 5). In comparison to unlesioned contralateral hemispheres, treated hemispheres of both the saline and VH14*PEST cohorts showed statistically insignificant increases in total microglial density, as measured stereologically (22 and 13%, respectively) (Fig. 5b). However, NbSyn87*PEST treatment induced a 63% increase in microglial density compared to controls (Fig. 5b). Consistent with these findings, microglia in the SN of NbSyn87*PEST-treated animals appear amoeboid in morphology, generally indicative of phagocytic macrophage activity (Fig. 5a, inset, red arrows). By contrast, observations of microglial morphology for saline-treated and VH14*PEST-treated animals preserve the canonical ramified, inactive shape (Fig. 5a, inset). These assessments implicate NbSyn87*PEST as a potential inducer of inflammatory response, although further investigation of glial surface markers and signal transduction is necessary to reveal a true immune response.

## Discussion

Currently, there are no disease-modifying clinical therapies being used directly to combat the synucleinopathy central to PD pathogenesis. Numerous recent efforts have aimed to halt pathogenic cellular propagation of fibrillar species through immunotherapy with promising success.^13–16,29^ However, immunotherapeutic modalities fail to adequately address the intracellular pathogenic cascade. Improper α-syn nucleation and oligomerization at axon terminals are central to mechanistic implications of PD pathology.^30^ Clearance of extracellular α-syn deposits may not actively prevent stochastic intracellular seeding and degenerative pathway activation.^31,32^ Furthermore, active pathways employed by therapeutic biologics for α-syn clearance may be functionally perturbed in PD cellular environments.^33–35^ Thus, the prevention of intracellular aggregation and maintenance of intracellular concentrations is critical in reversal of neurotoxicity.

We have previously shown that both VH14*PEST and NbSyn87*PEST were effective in reducing excess α-syn accumulation and limiting α-syn-mediated neurotoxicity in vitro.^22^ Proteins containing a PEST motif are often targeted for proteasomal degradation. Fusion of the PEST degron to anti-huntingtin intrabodies targets them and their intracellular cargo to the proteasome for degradation based on proteasome inhibition with specific proteasome inhibitor epoxomicin.^27^ Additionally, scrambling the PEST motif successfully eliminates the clearance of both the intrabody and its intracellular cargo.^27^ These findings have also been replicated with the VH14*PEST and NbSyn87*PEST constructs used in this study.^22^ For these targets to be degraded through the proteasome, they must remain soluble,^36^ which intrabody binding facilitates. When binding to the proteasome the cargo is degraded along with the short (<200 amino acids) nanobody. Although it is difficult to resolve the relative contributions of intrabody interference of target epitopes or proteasomal substrate clearance in the observed anti-amyloidogenesis effect of this study, our in vitro data leads us to hypothesize PEST-mediated α-syn clearance to be an essential factor in disaggregation.

Addition of the PEST degron to both nanobody aptamers enhanced hydrophilicity and decreased overall molecular charge to stabilize soluble constructs, allowing for superior α-syn targeting and proteosomal clearance.^26^ Consistent with these data are our findings of intrabody-mediated α-syn clearance and diminution of pathologic α-syn labeling in the SN (Fig. 2a, b). With Thioflavin-S staining of β-sheet aggregation, we confirmed p-S129-labeled species to be indicative of toxic aggregates and a representative marker for histopathology (Fig. 2c). Due to limited tissue material we were unable to appropriately quantify reductions in Thioflavin-S staining by stereology, although we were able to qualitatively observe reductions in aggregation in both intrabody-treated cohorts. We determined in previous in situ characterizations of these two candidate intrabodies that although NbSyn87*PEST clears α-syn more effectively than VH14*PEST, VH14*PEST modifies cellular stress associated with multi-factorial proteostasis to a greater extent, particularly in the case of α-syn promotion of mutant huntingtin proteinopathy.^22^ This aspect of intervention is integral for synucleinopathies that are commonly associated with concomitant pathology of additional proteins vulnerable to disordering or misfolding such as tau, amyloid-β, and mutant huntingtin. Abating proteostatic stress in PD, where both chaperone-mediated autophagic and macro-autophagic systems are perturbed and changes in cellular oxidation accelerate misfolding, may be a complementary clinical target to α-syn toxicity.^5,30,33,34^

This study aimed to use intrabody treatment to modify synucleinopathy in the context of canonical Parkinsonian motor pathology. Nigrostriatal pathology in PD is a complex process that is difficult to accurately and temporally model. Clinical PD begins as an axonopathy in which striatal terminal depletion and subsequent neurotransmittive failure provokes phenotypic onset.^37–39^ One of the α-syn’s primary roles in striatal axonopathy is phenotypic insult through synaptic injury.^37–41^ To that end, our model represents early PD-like pathology in which striatal innervation loss can clearly be observed through reductions in TH and DAT immunoreactive densities and depleted levels of striatal dopamine (Fig. 3). These deficits were directly correlated to α-syn aggregation that featured classical β-sheet amyloid organization (Thioflavin-S) and was resistant to Proteinase K digestion, validating the vector-induced accumulation as authentic Lewy body-like deposition (Fig. S2). Although our model did not feature nigral neuron loss at the time of animal sacrifice, the striatal deficits that were observed serve as early hallmarks of pathology and most likely precede downstream nigral neurodegeneration. Furthermore, evidence from the VH14*PEST cohort demonstrates that intrabody treatment can directly counteract and rescue nigrostriatal terminals from the early-phase, PD-like pathology observed in the saline-treated animals.

It is unclear why, although both intrabodies successfully remediated pathogenic aggregation of α-syn, VH14*PEST showed superior histological conservation of the nigrostriatal pathway, featuring protection of striatal integration, preserved expression of striatal DAT, and trending conservation of dopaminergic nigral neurons and striatal dopamine supply. One explanation may be the increased inflammatory signaling observed in the NbSyn87*PEST-injected SN which may exacerbate α-syn-induced pathology or counteract the expected effects of reduced α-syn aggregation.^42–44^ The minor increases in Iba-1-labeled cells in the saline and VH14*PEST-treated cohorts may be attributed to synuclein-mediated inflammation. The derivation of both nanobodies from different host species, human and camelid, provides a potential explanation in the divergent inflammatory reactions observed through microglial dynamics. The lower inflammatory response observed in the VH14*PEST group, combined with its human origin, suggests that this nanobody construct may be a more conservative therapeutic option. Stabilized inflammatory activation in the VH14*PEST treatment group also implies that viral delivery did not elicit nigral inflammation ipsilateral to injection. These data from the VH14*PEST cohort also validate that repeated injection with AAV5 virus alone does not induce stable, long-term inflammation. However, further analysis of microglial surface markers and monocyte expression is necessary to characterize any induction of active inflammation or potentiatiation of an immune response. Interestingly, NbSyn87*PEST treatment showed a modest improvement over VH14*PEST in functional preservation in behavioral assays, in contrast to indications of ineffectual nigrostriatal dopaminergic preservation. These findings may be a result of pronounced variability in individual animal performance in both the cylinder test and the stepping test and may necessitate a lengthier lesion procedure for future experimental iterations or deeper investigation of fine motor coordination, such as gait analysis.^45^

An exciting attribute of intrabody therapy is the use of viral vector delivery systems as a primary modality for long-term treatment. The use of AAV vectors to deliver long-term, recombinant payloads to combat neurological disease may be ideal for prolonged, age-related neurodegeneration.^46–48^ Potential cell replacement strategies for PD treatment may still require anti-α-syn therapeutic strategies, as toxic α-syn species have been shown to propagate into cell graft material.^11^ Intrabodies transduced into these cells pre-transplantation could protect grafted cells from host-transmitted synucleinopathy. However, a primary concern with gene therapy intrabody treatment, particularly with the deployment of proteosomal clearance mechanisms, is the effective titer and dosing paradigm required to reverse abnormal physiological concentrations of monomeric α-syn. Although early studies suggested minimal neurophysiological importance of α-syn expression,^49^ current data suggest that subthreshold knockdown of α-syn can induce dose-dependent neurodegeneration and functional decline in neurotransmission.^50,51^ Intrabody binding to α-syn may also interrupt physiological functional capacities, primarily in membrane-binding domains critical for vesicular trafficking and SNARE (soluble *N*-ethylmaleimide-sensitive factor activating protein receptor) complex arbitration.^52,53^ NAC region binding, as is the case for VH14*PEST, may be particularly conducive to loss-of-function α-syn interference in overdosed, prolonged expression systems. Our analysis of VH14*PEST, delivered at a high titer of 1 × 10^13^ vg/ml, revealed no exacerbation of α-syn phenotypic insults. Furthermore, the lack of statistically significant decrease in human-specific α-syn species, as denoted by LB509^+^ labeling (Fig. S1), suggests that the expression of VH14*PEST and NbSyn87*PEST is adequate for pathologic prevention based on the degree of α-syn overexpression in this model and maintains supra-threshold α-syn concentrations for proper function. These findings minimize concern of the potential for viral “overshoot” that may disproportionately induce excessive and detrimental clearance of α-syn, thus allowing for future studies exploring alternative delivery methods and higher-order model systems.

To date, nanobody technology has not been prominently utilized in in vivo settings. Although antibody fragments are high-affinity binders that optimize target specificity, in vitro aptamer stability has been difficult to reproduce in the cellular milieu of live animal models. The use of ER-tethered nanobodies to specifically halt transduction of protein products through the secretory pathway has shown early promise in loss-of-function targeting of toxic proteins in the context of Alzheimer’s disease and tumor-induced angiogenesis.^54^ Through our use of the PEST motif, we were able to stabilize our constructs in a reductive cellular environment to potentially overcome this hurdle while maintaining cytosplasmic scavenging potential. Perhaps the most frequent application of in vivo nanobody utilization has been in nanobody-assisted tumor-imaging modalities for cancer-specific cellular markers.^54–56^ However, effective use of nanobody technology in direct target protein interference has been slow. A recent report has shown nanobody efficacy in anti-fibrillarization of circulating β_2_-microglobulin in transgenic mice modeling genetic forms of hemodyalisis, although these nanobodies were targeting circulating protein aggregation.^57^ Our study is among the first to employ a gene therapy intrabody system that can specifically target amyloidogenesis of cytosolic protein within the central nervous system parenchyma.

In the context of progressing intrabody therapy into translational studies, it is important to evaluate the model system in which this set of experiments was conducted, as well as the timeline of potential therapeutic intervention. Our use of the AAV-α-syn overexpression model was intended to determine the baseline efficacy of intrabody therapy on pathologic aggregation. However, clinical observation of disease is significantly delayed compared to pathogenic onset and proteopathic seeding events. A supplemental, clinically relevant experimental paradigm may also include a model utilizing pre-formed α-syn aggregates to mimic toxic cell-to-cell propagation dynamics, prion-like seed propensity, and optimize α-syn substrate clearance.^58,59^ Additionally, growing consideration for peripheral sources of α-syn deposition, in particular via the gut–brain axis, warrant consideration of a global therapeutic paradigm for intracellular α-syn intervention.^60–62^ Our study design has served as a proof-of-principle investigation into nanobody perturbation of modeled PD pathophysiology, primarily nigrostriatal neurotoxicity. However, clinical PD, particularly α-syn centric pathology, occurs throughout the neuraxis with prevalent manifestation of extra-motor phenotypes. Recent reports illuminating advanced transduction efficiency throughout the central nervous system and periphery via engineered AAV capsids open the door for intrabody targeting of widespread α-syn pathology potentially through intrathecal or intravenous administration, enhancing the translational potential for clinical paradigms.^63^ The data presented in this study, in conjunction with extracellular protein clearance mechanisms, highlight an intriguing avenue for disruption of α-syn toxicity and targeted relief of proteostatic insult in neurological disease.

## Materials and methods

### Animals

All care and husbandry of animal subjects was carried out in accordance with protocols approved by the Institutional Animal Care and Use Committee at Rush University Medical Center. Forty-eight, 8-week-old Sprague–Dawley rats (Harlan Biotechnology) were maintained under a 12-hour light–dark cycle at 25 °C and 50% humidity. Animals were fed ad libitum chow and water. All animals used in the study were acclimatized in the testing facility for 1 week prior to initiation of experimentation.

### Behavioral assays

Rodent behavioral tests were performed as previously described.^64^ Modified cylinder tests and stepping tests were performed 1 week prior to lesioning and 2 weeks post lesion to establish baseline performance scores. Animals were pre-sorted into treatment groups based on post-lesion performance scores. Behavioral assays were conducted bi-weekly after treatment injections until the study end date after week 17. Performance for both tests was assessed by analyzing the percentage of forelimb contacts contralateral to the side of lesion.

### Stereotaxic surgery

All stereotaxic surgical procedures were followed as previously described.^65^ Animals were anesthetized with sodium ketamine hydrochloride (100 mg/kg)/xylazine hydrochloride solution (10 mg/kg). All subjects were lesioned via unilateral injections of AAV5-α-synuclein (volume: 2 μl, vector titer: 1 × 10^12^ vg/ml, UNC Vector Core) into the right substantia nigra (Coordinates: A/P at −4.8 mm, M/L at 1.7 mm, and D/V at −7.5 mm) at a flow rate of 0.2 μl/min. Animals were sorted into three treatment groups based on post-lesion behavioral performance, and a second round of surgeries were performed 3 weeks post lesion. Groups 1 and 2 received intranigral injections (volume: 2 μl, vector titer: 1 × 10^13^ vg/ml, UNC Vector Core) at a flow rate of 1.0 μl/min of AAV5-VH14*PEST and AAV5-NbSyn87*PEST, respectively. Group 3 received an injection of vehicle saline at the same injection conditions.

### Rodent necropsy and tissue processing

At the study endpoint, animals were anesthetized with and perfused transcardially with 0.9% sterile phosphate-buffered saline (PBS) solution or 4% paraformaldehyde solution at 4 °C. Brains perfused with PBS were snap frozen for high-performance liquid chromatography (HPLC) processing. Brains perfused in 4% paraformaldehyde were post-fixed overnight in 4% paraformaldehyde solution and subsequently transferred to a 10, 20, and 30% sucrose gradient until the tissue sank. Brains were maintained in sucrose solution until they were cut on a freezing-stage sliding microtome into 40 μm coronal sections. Sections were stored in cryopreservative solution until immunohistochemical processing.

### High-performance liquid chromatography

All HPLC protocols were followed as previously described.^66^ Striata from eight animals in each cohort were extracted, processed, and analyzed for concentrations of dopamine. Tissue was sonicated in 0.2 M perchloric acid with isoproterenol. Centrifuged supernatant was injected in a Eicompak SC-3ODS column (JM Science, Inc.) and analyzed as per the manufacturer’s protocol.

### Immunohistochemistry, Proteinase K, and Thioflavin-S staining

All immunohistochemistry protocols were followed as previously described.^65^ Free-floating sections (40 μm) of coronal brain slices were stored in cryoprotectant and quenched with sodium periodate prior to staining. Sections for immunohistochemistry were incubated with the primary antibodies listed in Table S1. Sections were subsequently incubated with the respective biotinylated secondary antibodies (Table S1) and labeled with a standard ABC HRP Biotin/Avidin Complex Kit (Vector Laboratories). Staining was developed with DAB and sections were mounted for imaging and analysis. Proteinase K digestion protocol was followed as previously described.^65^ Briefly, sections were mounted and dried on gelatin-coated slides. Tissue was rehydrated and incubated with 10 μl/ml of Proteinase K (Invitrogen) dissolved in TBST for 30 min at 55 °C. Sections were subsequently fixed with 4% paraformaldehyde for 10 min and immunostained for α-syn as previously described. Preceding Thioflavin-S staining, tissue was pre-processed for phosphorylated S129 α-syn immunofluorescence using adapted staining protocol previously described. Tissue was mounted and dried on gelatin-coated slides at room temperature. Sections were defatted in chloroform for 2 h and hydrated through graded alcohols. Tissue was incubated in 0.1% Thioflavine-S solution (Sigma) for 10 min prior to imaging. All immunofluorescent images were taken on an Olympus confocal laser scanning microscope with the Fluoview software.

### Densitometry

Optical density measurements were taken using Scion Image (v1.63, Meyer Instruments). Samples were imaged on an Olympus BH-2 microscope (Olympus) and corresponding regions imaged were aligned in a reading frame prior to recording. Densitometric measurements from a minimum of two sections in a half-series (every 12th section) of each subject were recorded and averaged for quantitative measurements. Data from variable regions were normalized with in-subject, uninjected control hemispheres.

### Stereology

Cell counts were estimated by unbiased stereological counting methods using the optical fractionator probe in StereoInvestigator (v10.40, MBF Biosciences).^67^ All raters were blinded to subject ID prior to analysis. Sections were viewed on an Olympus BX51 microscope (Olympus) with specific brain region contours detected and traced at ×4 objective and data recordings conducted with ×60 magnification. Serial sections of biological replicates were recorded using every 12 sections of 40 μm coronal slices. Stereological assessments of TH^+^-labeled cells were used to establish degree of lesion affecting dopaminergic neurons in the substantia nigra. To assess quantitatively the level of inflammation at the site of a given lesion, Iba-1^+^-labeled cells were also counted in the substantia nigra.

### Statistical analysis

All data are presented as mean ± SEM. All statistical analyses were performed using the GraphPad Prism (version 7.03). Statistical differences were determined by one-way or two-way analysis of variance, with corrections for repeated measures as needed. Specific statistical analyses and significant findings are noted in figure legends.

### Data availability

All relevant data and analyses from this study are available from the corresponding author upon request.

## Electronic supplementary material

Supplementary Information

## Electronic supplementary material

**Supplementary information** accompanies the paper on the *npj Parkinson’s Disease* website (10.1038/s41531-018-0062-4).
