# Supplementary material for: Proteasome-targeted nanobodies alleviate pathology and functional decline in an α-synuclein-based Parkinson’s disease model
Source: NPJ Parkinsons Dis. 2018 Aug 22;4:25. doi: 10.1038/s41531-018-0062-4 (PMC6105584; doi:10.1038/s41531-018-0062-4)
Supplement: Supplementary file 1 — Supplementary Information [file 41531_2018_62_MOESM1_ESM.pdf]

Proteasome-targeted nanobodies alleviate pathology and functional decline in an α-synuclein-based Parkinson's disease model

Diptaman Chatterjee[1], Mansi Bhatt[1], David Butler[2], Erwin De Genst[3], Christopher M. Dobson[3], Anne Messer[2], Jeffrey H. Kordower[1,4]

[1] Department of Neurological Sciences, Rush University Medical Center, Chicago, Illinois 60612

[2]Neural Stem Cell Institute 1 Discovery Dr. Rensselaer, New York, 12144 and Department of Biomedical Sciences; University at Albany, Albany, NY, 12208

[3]Centre for Misfolding Diseases, Department of Chemistry, University of Cambridge, CB2 1EW Cambridge, United Kingdom

[4] Van Andel Research Institute, Grand Rapids, Michigan 49503

Address all correspondence to:

Jeffrey H. Kordower Ph.D.

Department of Neurological Sciences

Rush University Medical Center

1735 West Harrison Street

Chicago Illinois 60612

312-563-3585 (tel)

312-563-3571 (fax)

jkordowe@rush.edu

Supplemental figures

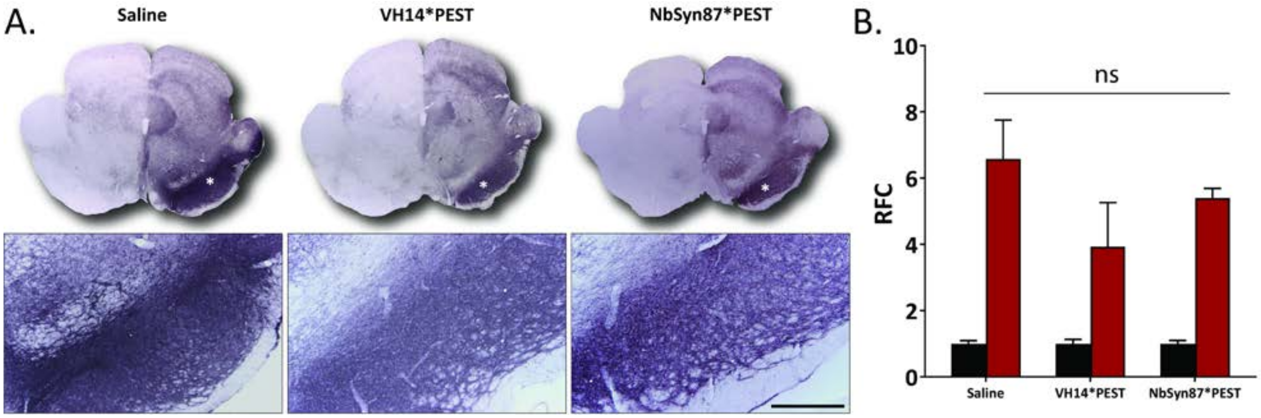

Figure S1.

α-Syn expression levels in the substantia nigra are not significantly reduced post-intrabody treatment. **A)** Immunolabeling of human specific α-syn (LB509) in the substantia nigra. **B)** Densitometric quantification of LB509 labeling reveals decreases in intrabody treated-cohorts, but not at statistically significant levels. The data were analyzed via one-way ANOVA with Tukey post-hoc comparisons (n=7 for all groups).

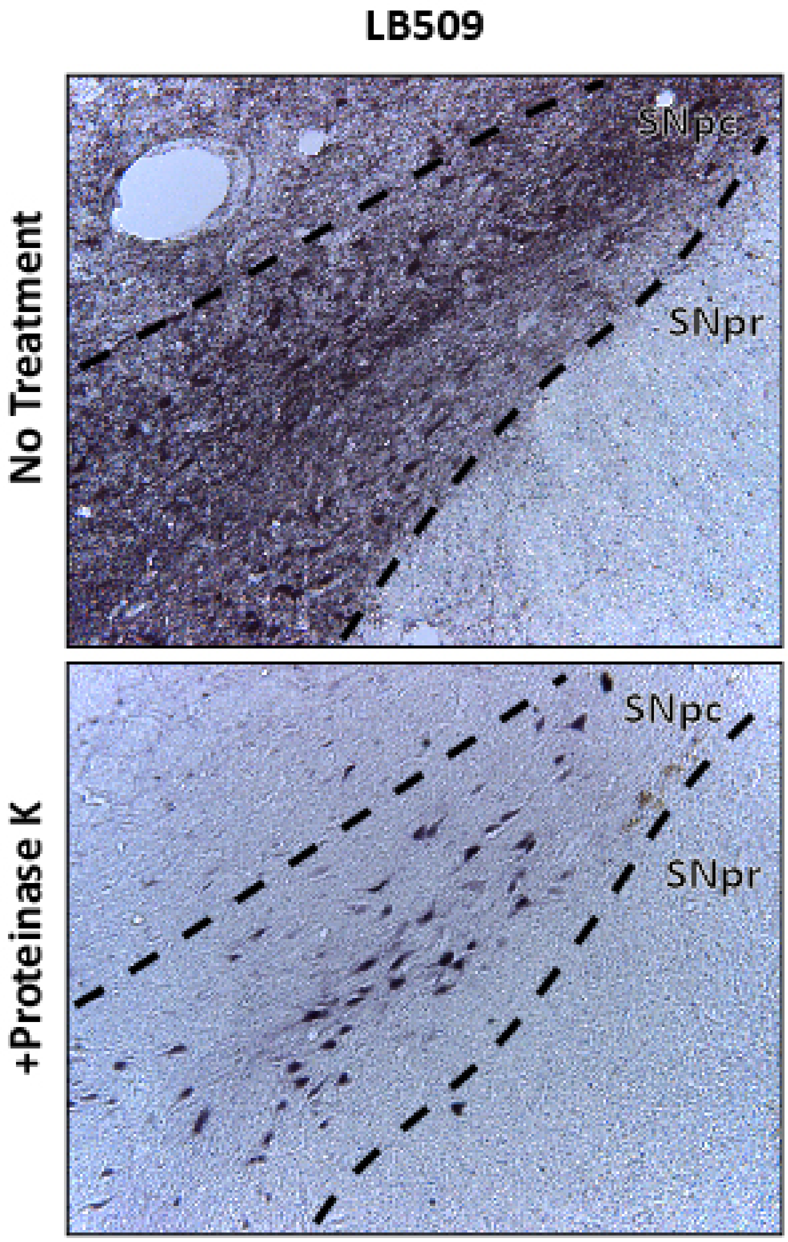

Figure S2.

AAV5-α-Syn over expression in the substantia nigra pars compacta induces pathologic, insoluble Lewy-body like cellular inclusions that are resistant to proteinase K digestion.

**Antibody Type**	**Antibody**	**Species**	**Company**	**Catalog #**	**Dilution**
**Primary Antibody**	pS129 alpha-synuclein	Rabbit	Abcam	ab51253	1:500
	LB509 alpha-synuclein	Mouse	Thermo Fischer	180215	1:500
	Tyrosine Hydroxylase	Rabbit	Immunostar	202984	1:5000
	DAT	Rabbit	EMD Millipore	MAB369	1:200
	Hemagglutinin	Mouse	Covance	MMS-101P	1:1000
	Iba-1	Rabbit	Wako	019-19741	1:2500
**Secondary Antibody**	Biotin. Goat Anti-rabbit	Goat	Vector Labs	BA-1000	1:200
	Biotin. Horse Anti-mouse	Horse	Vector Labs	BA-2000	1:200

Table S1.

List of primary and secondary antibodies used for immunohistochemistry and analysis.
